# Genotoxin Induced Mutagenesis in the Model Plant *Physcomitrella patens*

**DOI:** 10.1155/2013/535049

**Published:** 2013-12-09

**Authors:** Marcela Holá, Jaroslav Kozák, Radka Vágnerová, Karel J. Angelis

**Affiliations:** ^1^Institute of Experimental Botany ASCR, Na Karlovce 1, 160 00 Praha 6, Czech Republic; ^2^Institute of Organic Chemistry and Biochemistry ASCR, Flemingovo nám. 2, 1600 00 Praha 6, Czech Republic

## Abstract

The moss *Physcomitrella patens* is unique for the high frequency of homologous recombination, haploid state, and filamentous growth during early stages of the vegetative growth, which makes it an excellent model plant to study DNA damage responses. We used single cell gel electrophoresis (comet) assay to determine kinetics of response to Bleomycin induced DNA oxidative damage and single and double strand breaks in wild type and mutant *lig4 Physcomitrella* lines. Moreover, *APT* gene when inactivated by induced mutations was used as selectable marker to ascertain mutational background at nucleotide level by sequencing of the *APT* locus. We show that extensive repair of DSBs occurs also in the absence of the functional LIG4, whereas repair of SSBs is seriously compromised. From analysis of induced mutations we conclude that their accumulation rather than remaining lesions in DNA and blocking progression through cell cycle is incompatible with normal plant growth and development and leads to sensitive phenotype.

## 1. Introduction

Plants developed several strategies to protect integrity of their genome against various environmental stresses. Common denominator of most of them is oxidative stress mediated by reactive oxygen species (ROS). The origin of ROS within the cell could be a consequence of physical or chemical genotoxic treatment, as well as byproduct of internal oxygen metabolism often triggered by external stimuli as drought and salinity. To be able to cope with oxidative stress we have to assess all faces of this challenge for plants; in particular, how it affects genetic material of the cells and how eventual changes are temporarily or permanently expressed in plant phenotype. This is why we need a flexible and robust model system, which experimentally enables the use of reverse genetics for genotoxic and biochemical studies. In this paper we describe a novel system to be considered for genotoxicity testing in plants.

The moss *Physcomitrella patens* is an emerging model plant [1] with the following differences/advantages as compared to other plant test systems: efficient homologous recombination (enabling reverse genetics of virtually any gene), dominant haploid phase (enabling assessment of mutation phenotype), small size plantlets colonies with a quick and during early vegetative stage also filamentous growth, easy cultivation in inorganic media and several options of long term storage. Here we describe and validate a system of small protonemata fragments with high fraction of apical cells primarily developed for the purpose of genotoxicity testing. However, these one-day-old protonemata could be used as a substitute of protoplasts for other purposes, for example, for moss transformation [2].

APT (adenine phosphoribosyltransferase) is an enzyme of the purine salvage pathway that converts adenine into AMP and its loss of function generates plants resistant to adenine analogues, for example, 2-FA (2-Fluoroadenine) [3]. Mutational inactivation can be used as selectable marker for mutator genotyping as well as analysis of mutations in *APT *locus on nucleotide level [4–6].

This paper is an extension of our previous study of *Physcomitrella* knockout mutants of a key MRN (MRE11, RAD50, and NBS1) complex [6] with a pleiotropic effect on DSB repair in whole. We explore and validate the above outlined moss model system for genotoxicity testing in plants. We describe a parallel use of SCGE (single cell gel electrophoresis, comet) assay for detection of DNA damage and its repair and of APT assay with sequencing analysis of mutants. On example of *lig4*, mutated in a key component of nonhomologous DSB-end joining pathway (C-NHEJ), we show consequences of mis repair. For strengthening the model concept we also present preliminary results of *pprad51AB* sensitivity to genotoxin treatment and on *ppku70* mutation rate.

## 2. Material and Methods

### 2.1. Plant Material

*Physcomitrella patens* (Hedw.) B.S.G. “Gransden 2004” wild type and *pplig4 *were vegetatively propagated as previously described [7]. The *lig4 *and *ku70* mutants of C-NHEJ were generated by D. G. Schaefer, Neuchatel University, Switzerland, and F. Nogue, INRA, Paris, France, and kindly provided by F. Nogue. Detailed characteristic of this mutant will be published elsewhere. Mutant in both alleles of *Physcomitrella RAD51* gene (*pprad51AB*, clone 721) is described elsewhere [8] and was kindly provided by B. Reiss, MPIZ, Cologne, Germany.

Individual plants were cultured as “spot inocula” on BCD agar medium supplemented with 1 mM CaCl_2_ and 5 mM ammonium tartrate (BCDAT medium) or as lawns of protonemal filaments by subculture of homogenized tissue on BCDAT agar medium overlaid with cellophane in growth chambers with 18/6 hours day/night cycle at 22/18°C.

One-day-old protonemal tissue for repair and mutation experiments were prepared from one-week-old tissue scraped from plates, suspended in 8 mL of BCD medium, and sheared by a T25 homogenizer (IKA, Germany) at 10 000 rpm for two 1-minute cycles and let 24 hours to recover in cultivation chamber with gentle shaking at 100 rpm. This treatment yielded suspension of 3–5 cell protonemata filaments, which readily settle for recovery. Settled protonemata could be handled without excessive losses by tweezers on glass Petri plates.

### 2.2. Bleomycin Treatment and Sensitivity Assay

For treatments was used Bleomycin sulphate supplied as Bleomedac inj. (Medac, Hamburg, Germany). All solutions were prepared fresh prior treatment from weighted substance in BCDAT medium.

Protonemal growth was tested by inoculating explants of wild type and 5 mutant lines onto 6 × 4 multiwell plates organized to allow in line comparison of the effect of increasing Bleomycin concentrations. The wells were filled with 2 mL of standard growth BCDAT agar medium without or with 0.01, 0.1, and 1 *μ*g mL^−1^ Bleomycin. The experiment was carried in 3 independent replicas and monitored up to 3 weeks for growth of “spot inocula.”

Treatment of one-day-old protonemata was performed on glass 5 cm Petri plates with the aid of bent tweezers to handle tissue and pipettes to remove excess liquids. Opening of yellow tips is generally small enough to avoid suction of moss filaments when drawing majority of liquid from tissue prior blotting of collected tissue on filter paper to remove the rest.

In dose-response and repair kinetic experiments, one-day-old protonemata were after the Bleomycin treatment thoroughly rinsed in water, blotted on filter paper, and either flash-frozen in liquid N_2_ (dose response and repair *t* = 0) or left to recover on plates in liquid BCDAT medium for the indicated repair times, before being frozen in liquid N_2_.

For induction and regeneration of *apt* mutants one-day-old protonemata were after Bleomycin treatment thoroughly rinsed with H_2_O, suspended in 2 mL of BCDAT medium, and spread on cellophane overlaid BCDAT agar plates, which were for selection supplemented with 2-FA (2-Fluoroadenine, Sigma-Aldrich, cat. Nr. 535087) and further incubated in growth chamber.

### 2.3. Detection of DNA Lesions

DNA single and double strand breaks were detected by a SCGE assay using either alkaline unwinding step A/N [9, 10] or fully neutral N/N protocol [11, 12] as previously described. In brief, approximately 100 mg of frozen tissue was cut with a razor blade in 300 *μ*L PBS + 10 mM EDTA on ice and released nuclei transferred into Eppendorf tubes on ice. 70 *μ*L of nuclear suspension was dispersed in 280 *μ*L of melted 0.7% LMT agarose (GibcoBRL, cat. Nr. 15510-027) at 40°C and four 80 *μ*L aliquots were immediately pipetted onto each of two coated microscope slides (in duplicate per slide), covered with a 22 × 22 mm cover slip and then chilled on ice for 1 min to solidify the agarose. After removal of cover slips, slides were immersed in lysing solution (2.5 M NaCl, 10 mM Tris-HCl, 0.1 M EDTA, and 1% N-lauroyl sarcosinate, pH 7.6) on ice for at least 1 hour to dissolve cellular membranes and remove attached proteins. The whole procedure from chopping tissue to placement into lysing solution takes approximately 3 minutes. After lysis, slides were either first incubated 10 minutes in 0.3 M NaOH, 5 mM EDTA, pH 13.5 solution to allow partially unwind DNA double helix to reveal SSBs (A/N protocol) or without unwinding step (N/N protocol) directly equilibrated twice for 5 minutes in TBE electrophoresis buffer to remove salts and detergents. Comet slides were then subjected to electrophoresis at 1 V cm^−1^ (app. 12 mA) for 3 minutes. After electrophoresis, slides were placed for 5 min in 70% EtOH, 5 min in 96% EtOH, and air-dried.

Comets were viewed in epifluorescence with a Nikon Eclipse 800 microscope stained with SYBR Gold (Molecular Probes/Invitrogen, cat. Nr. S11494) according to manufacture recommendation and evaluated by the LUCIA Comet cytogenetic software (LIM Inc., Czech Republic).

### 2.4. SCGE Assay Data Analysis

The fraction of DNA in comet tails (% tail-DNA, % T DNA) was used as a measure of DNA damage. Data for the wild type and the mutant *pplig4* line analysed in this study were obtained in at least three independent experiments. In each experiment, the % T DNA was measured at seven time points: 0, 5, 10, 20, 60, 180, and 360 min after the treatment and in control tissue without treatment. Measurements included four independent gel replicas of 25 evaluated comets totalled in at least 300 comets analysed per experimental point. The percentage of damage remaining as plotted on Figure 2(b) after given repair time (*t*_*x*_) is defined as

(1) %  damage  remaining  (tx)=mean  %  tail-DNA  (tx)−mean  %  tail-DNA  (control)mean  %  tail-DNA  (t0)−mean  %  tail-DNA  (control) ∗100.

Time-course repair data were analysed for one- or two-phase decay kinetic by Prism v.5 program (GrafPad Software Inc., USA).

### 2.5. Isolation and Analysis of *apt* Mutants after Bleomycin Treatment

Mutation rates were measured as the number of *apt* mutants that appeared as green foci of regenerating clones resistant to 2-FA (Figure 3). Treated protonemata were first incubated approximately 3 weeks on plates with 2 or 3 mM 2-FA until first green foci start to emerge. Then whole cellophane overlay was transferred to a new plate with 8 mM 2-FA and emerging clones were allowed to form colonies. Stable clones were then counted.

Some clones were further propagated on plates with 8 mM 2-FA and their *APT* locus was subsequently PCR amplified and sequenced to identify the mutation(s) responsible for the resistance. Approximately 100 mg of tissue was used to isolate genomic DNA with DNeasy Plant Mini Kit (Qiagen, cat. Nr. 69104) using “ball” mill Retsch MM301 to homogenize the tissue in 2 mL round bottom Eppendorf tubes. *APT* locus was amplified from isolated genomic DNA with KOD Hot Start DNA Polymerase (Millipore/Novagen, cat. Nr. 71086), purified with the QIAquick PCR Purification Kit (Qiagen, cat. Nr. 28104) and used as a template for sequencing with BigDye Terminator v3.1 Cycle Sequencing Kit (Applied Biosystems, cat. Nr. 4337455). Locations of PCR primers used for *APT* amplification and sequencing are depicted on Supplementary Figure 2 and their sequences are listed in Supplementary Table 1 (see Supplementary Material available online at http://dx.doi.org/10.1155/2013/535049). To keep sequencing cost down only half volume of the BigDye Ready Reaction Mix was used in a standard sequencing reaction and combined with the same volume of Half-Term-Dye-Termination mixture (Sigma-Aldrich, cat. Nr. H1282).

### 2.6. Analysis of Sequencing Data

Sequences of each clone obtained on genetic analyser Prism 3130x1 (Applied Biosystems, USA) were stiched together with MacVector program Assembler 12.7.5 (MacVector, USA) into contigs and aligned to the latest annotated *APT* sequence Pp1s114_124V6.1 in the COSMOSS—the *Physcomitrella patens* resource database (https://www.cosmoss.org/).

## 3. Results and Discussion

In all experiments a model *Physcomitrella patens* has been used as one day recovered fragments of 3–5 cell size derived from the lawn of growing protonema filaments by extensive shearing. Such one-day-old protonemata represent a unique system among plants to study plant tissue with up to 50% of apical dividing cells. Convenient mechanical handling enables quick processing of tissue after the treatment to address short repair times and with fine tip tweezers also for uniform spotting to test sensitivity. In this respect one-day-old protonemata are preferred system to so far widely used protoplasts, which could be collected only by centrifugation. Another reason for using protonemata is possibility their mechanical disintegration by razor blade chopping for rapid release of nuclei for SCGE assay. In this way direct use of protoplasts for comet assay is obstructed by nearly instant regeneration of the cell wall within 4 hours after the release from cellulase treatment (unpublished observation), because cell wall prevents DNA movement from nuclei during electrophoresis.

### 3.1. Sensitivity to Bleomycin Treatment

**Figure 1:**
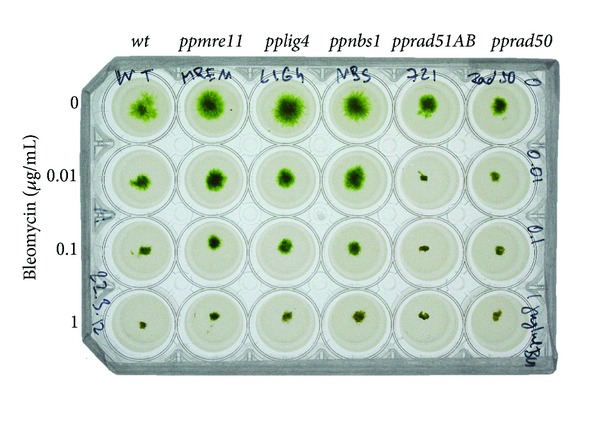
Sensitivity of the *Physcomitrella patens *repair mutants *mre11, lig4, nbs1, rad51AB, *and* rad50* to chronic exposure of Bleomycin. *Physcomitrella* explants were inoculated as “spot inocula” onto BCDAT-agar plates supplemented with 0, 0.01, 0.1, and 1 *μ*g mL^−1^ Bleomycin and photographed 10 days after inoculation.

Moss wild type and *pplig4*, *mre11*, *nbs1*, *rad51AB*, and *rad50* [6, 8] mutant lines were analysed for their sensitivity to radiomimetic Bleomycin in chronic “survival” assay when test plates with various concentrations of Bleomycin were inoculated with equal tissue “spots” of one-day-old protonemata and incubated up to 3 weeks (Figure 1). Only *rad51AB* and *rad50* strains displayed one order of a magnitude higher sensitivity in comparison to other tested lines. The survival growth of *ppmre11* is somehow in contradiction with pervious results of Kamisugi et al. [6], but one has to realize different assay conditions, for example, acute versus chronic exposure and protoplast cells versus protonemata. In protonema tissue under permanent genotoxic stress *mre11* express phenotype similar to wild type, *nbs1*, and also *lig4*. One can speculate that 3′ to 5′ exonuclease and endonuclease activity associated with MRE11 is dispensable for tissue survival, but proteins RAD50 and RAD51 supporting DNA structure are not. Kozak et al. [12] previously showed crucial role of structural maintenance of chromosome complex SMC5/6 in the repair of Bleomycin induced DSBs. In this context RAD50s have similar structural role in MRN complex as SMCs in the structure of the SMC5/6 complex [13]. Both these complexes can function in tethering of broken ends in close proximity.

### 3.2. Induction of DNA Lesions and Their Repair

Bleomycin, an ionizing radiation mimicking agent, functions as a catalyst activated by interaction with DNA and attachment of Fe^2+^ to produce oxygen radicals leading to lesions as SSBs, DSBs, AP-sites, and damaged bases [14, 15], which all could be readily detected by SCGE [16]. DNA breaks and other lesions converted to breaks lead to DNA fragmentation and nucleoid unwinding allowing relaxed DNA to move in electric field from nuclei out to form a “comet” like object in which increased quantity of fragmented DNA in comet tail (% T DNA) is proportional to breakage. DSBs are detected by an N/N assay when pH of lysing and electrophoretic solutions is kept under pH 10, whilst for the detection of SSBs DNA after the lysis is allowed to unwind DNA double-helix in alkali [17] to separate individual strands and expose their fragmentation (A/N protocol [9]).

**Figure 2:**
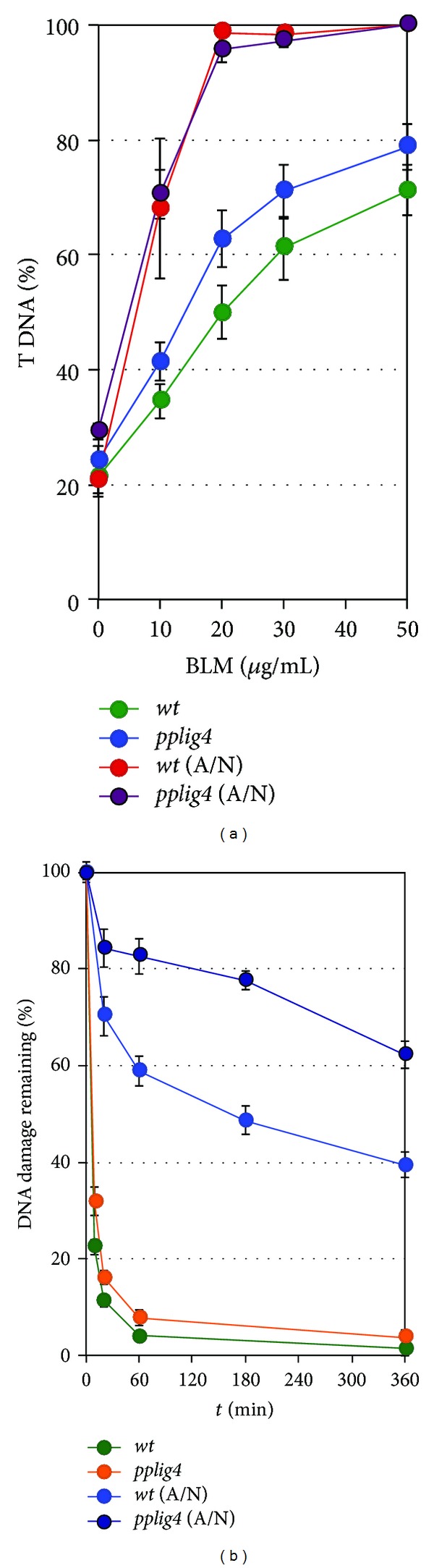
SSB and DSB repair kinetics determined by SCGE. One-day regenerated protonemal tissue from wild type and *pplig4* lines was treated with Bleomycin for 1 h prior to nuclear extraction and the analysis. (a) Dose-response as the percentage of the free DNA moved by electrophoresis into comet tail (% T DNA) at the indicated Bleomycin concentrations. DSBs were determined by N/N protocol: green: wild type, blue: *pplig4, *whereas SSBs were determined by A/N protocol: red: wild type, dark purple: *pplig4*. (b) Repair kinetics is plotted as % of DSBs remaining over the 0, 5, 10, 20, 60, 180, and 360 min period of repair recovery. Maximum damage is normalised as 100% at *t* = 0 for all lines. SSBs were induced by 1-hour treatment with 2 *μ*g mL^−1^ Bleomycin; bright blue: wild type, dark blue: *pplig4*, and determined by A/N protocol. DSBs were induced by 1-hour treatment with 30 *μ*g mL^−1^ Bleomycin, green: wild type, orange: *pplig4*, and determined by N/N protocol. (Error bars-standard error).

Bleomycin fragmentation of genomic DNA by induction of SSBs and DSBs is documented on Figure 2(a). Ten times higher efficiency to induce SSBs than DSBs is in agreement with generally accepted ratio of 1 : 10, DSBs versus SSBs, induced by ionizing radiation. Evidently this also applies for Bleomycin treatment of *Physcomitrella*. The background level of genomic DNA damage in wild type and *pplig4* is similar, between 20 and 25% T DNA, indicating that the repair defect has no significant effect on natural levels of genomic DNA fragmentation. Nevertheless, in comparison with wild type, *pplig4* is vulnerable to Bleomycin induction of DSBs and SSBs.

In both wild type and *pplig4* lines, the Bleomycin induced DSBs are repaired with a rapid, biphasic kinetics (Figure 2(b)). Half-lives of DSB survival *τ*_1/2_ 1.5 min for wild type and 2.5 min for *pplig4 *are similar to *τ*_1/2_ 2.9 min for *pprad50*, *τ*_1/2_ 4.1 min for* ppmre11*, and *τ*_1/2_ 1.9 min for *ppnbs1* previously reported in [6].

Contrary to DSBs, SSBs are repaired far less efficiently. Slow SSB repair might be common feature of plants since Donà et al. [18] recently observed similar phenomenon in *Medicago truncata* cell culture irradiated with different doses of *γ*-ray. The SSB repair kinetic in wild type *Physcomitrella* is clearly biphasic and in this respect parallels repair of MMS induced SSBs in *Arabidopsis* [19]. In comparison to DSBs, substantially smaller fraction of SSBs is repaired with fast kinetics; the defect even more manifested in *pplig4*. It suggests an important role for LIG4 in the repair of DNA lesions like modified basis, AP sites that are usually detected as SSBs and are repaired via BER (base excision repair). It is noteworthy that LIG3, the ligase finishing BER pathway, is not represented in plants. We showed earlier that principal substitute for LIG3 in *Arabidopsis* is LIG1 [19]. The repair kinetic of MMS induced SSBs in *atlig1* posed an exceptional route. After the treatment the number of breaks continues to increase during the first hour of repair and after 3 hours returns to the level at the end of treatment. Then repair continues similarly as in the wild type (see Figure 4 in [19]). Because *atlig1* is an *RNAi* line with only 40% of remaining LIG1 activity, such repair course is a consequence of unbalanced BER due to attenuated ligation step. Evidently the knockout mutation in *pplig4* does not have such severe effect on repair of SSBs; nevertheless, the defect clearly shows that LIG4 is also involved in the repair of SSBs in plants.

### 3.3. Induction and Analysis of *apt* Mutants

The mutator phenotype was assessed as the loss of function of the *APT* gene [4] due to presence or error prone repair of endogenous DNA damage in the wild-type moss and *lig4*, *mre11*, and *rad50* repair mutant lines.

**Figure 3:**
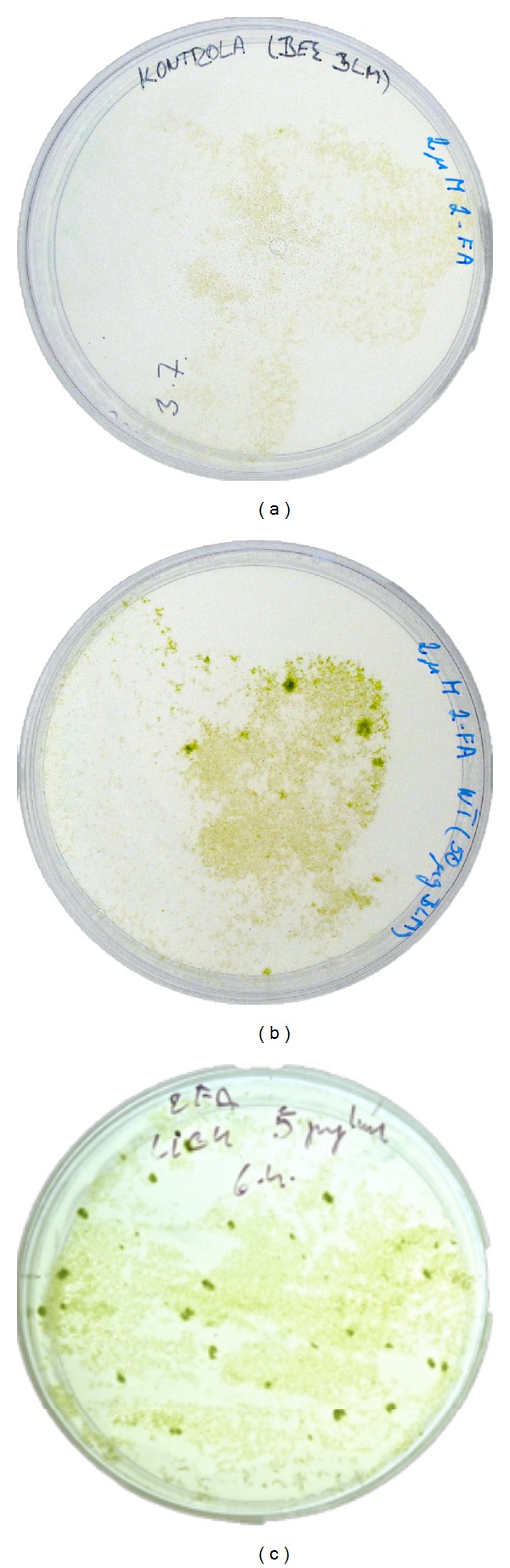
Plates with 2-FA resistant foci of wild type *Physcomitrella* (a and b) and *pplig4* (c) after 3 weeks of selection. (a) Untreated *Physcomitrella *wild type, (b) 50 *μ*g mL^−1^ Bleomycin treated wild type protonemata for 2 hours, and (c) 5 *μ*g mL^−1^ Bleomycin treated *pplig4* protonemata for 1 hour prior being spread on plates with BCDAT medium supplemented with 2 *μ*M 2-FA and cultivated for 3 weeks.

We found dramatic, over two orders of magnitude, variation of mutator phenotype in response to mutagenic treatment. While wild type *Physcomitrella* with low mutator phenotype needed 2 hours and 50 *μ*g mL^−1^ Bleomycin treatment to induce any *apt* clone, in *pprad50* with high mutator phenotype 1 hour treatment with only 0.1 *μ*g mL^−1^ Bleomycin was enough for massive induction of *apt *clones. Other lines, *pplig4* and *ppmre11*, assumed as having “moderate” mutator phenotype, were mutagenized either with 5 or, respectively, 1 *μ*g mL^−1^ Bleomycin for 1 hour. Mutagenesis and clone selection in *Physcomitrella* wild type and *pplig4* is depicted on Figure 3. For comparison we normalised the yield of 2-FA resistant clones to 1 *μ*g mL^−1^ Bleomycin treatment per 1 g dry tissue weight in each line as “relative number of *ppapts*.” The values of these normalised yields range from 9 in wild type to 875 for *pprad50 *(see Supplementary Figure 1 where are plotted summarized results of Bleomycin mutagenesis in *Physcomitrella* wild type and *lig4, ku70, rad50, mre11*, and *nbs1* mutants).

**Figure 4:**
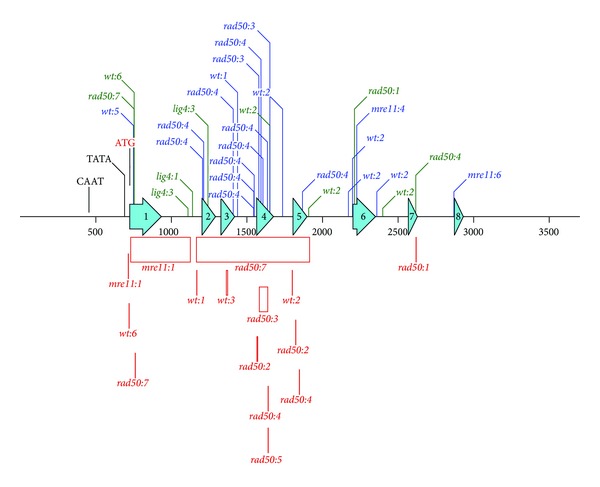
Map of identified mutations within *APT* locus. Bleomycin induced mutations are identified by color (blue: substitutions, green: insertions, and red: deletions) and tagged according to background as *wt*, *lig4*, *mre11*, and *rad50* and the number of line in which mutation was detected. Deletions are shown as boxes of size proportional to their length. On the locus map are depicted 500 nucleotide size markers and eight turquoise hollow arrows representing exons of *APT* CDS. Detailed description of each mutation is provided in Supplementary Table 2.

Randomly picked *apt* clones from selection plates were further propagated on 2-FA media to provide enough material for isolation of genomic DNA and sequencing analysis of *APT* locus. Results of sequencing analysis are pictured in Figure 4 and detailed annotations of identified mutations are summarized in Supplementary Table 2. In total were analysed 5 clones of *Physcomitrella* wild type, 4 clones of *pplig4*, 3 clones of *ppmre11*, and 6 clones of *pprad50* and identified 48 mutations. Mutations were according to assumed mechanism of formation classified as reversions, single base insertion or deletion, and insertions or deletions larger than 2 bases either in coding (exons) or noncoding regions of *APT* locus.

Most of the identified mutations are as expected localised within CDS, in particular within exon 4 that is annotated as coding for adenine salvage activity (see Figure 4). Nevertheless, in *wt:1*, *lig4:1*, *lig4:2*, and *mre11:6 apt *clones, mutations were identified only in the noncoding region and their contribution to mutated *APT* phenotype has to be established. Majority of mutations in CDS are point mutations (base substitution, single base insertions, and deletions) and it is difficult to dissect the route of their formation. Some of single base deletions could come from classical or altered NHEJ repair of DSBs [20], but more likely they represent along with other point mutations outcome of processing base oxidative damage. Interesting point is that only single base insertions were identified in *APT* CDS of *pplig4*. Insertion of extra base might imply defect in BER repair of oxidative damage in the absence of LIG4 and could be associated with defective repair of SSBs in *pplig4*.

Long deletions are clearly associated with NHEJ repair of DSBs, because, besides one rather short (8 bp) deletion in *wt:3* clone, all appear in clones derived from *mre11* and *rad50* background. This supports our working hypothesis that MRN-unsupervised repair generates more severe forms of genomic damage [6].

Only one 4 base insertion was identified in noncoding region of *wt:2*.

## 4. Conclusions

We validated the use of regenerating one-day-old protonemal tissue of *Physcomitrella patens* for complex analysis of genotoxic stress by parallel study of DNA damage, its repair, and mutagenic consequences in wild type and *lig4* mutant plants. From experimental point of view we developed a novel model system where 3–5 cell protonemata filaments with up to 50% of apical cells can substitute and surplus protoplasts use. Bleomycin was used to model DNA oxidative genotoxic stress with all its consequences as SSBs and DSBs, which can be followed by SCGE. We confirmed in *Physcomitrella* as previously in *Arabidopsis* rapid DSB repair even in the absence of LIG4, the key ligase of major DSB repair pathway by NHEJ mechanism [12]. Moreover, we showed crucial role of LIG4 in the repair of SSBs by BER mechanism, where it can substitute along with LIG1 [19] in plants missing LIG3. We selected and analysed by sequencing 2-FA resistant clones with Bleomycin mutated *APT* locus and found out that mutation spectra of *lig4* mutant reflects rather the defect of SSB than DSB repair. Nevertheless, as previously described [6], we interpret that mutations due to the error prone repair in *pplig4* rather than unrepaired lesions within DNA and interfering with progression through the cell cycle are responsible for *pplig4* sensitive phenotype.

## Supplementary Material

Supplementary Figure 1: Normalized mutation frequencies in Physcomitrella wild type and lig4, ku70, rad50, mre11and nbs1 mutant lines.
Supplementary Figure 2: Map and localization of APT primers used for rescue of APT locus and sequencing and which are listed in Supplemental Table1.
Supplementary Table 2: Mutations (location and description) identified in the APT locus of Physcomitrella wild type and lig4, mre11 and rad50 mutant lines.

## Abbreviations

A/N: Comet assay with alkaline unwinding step
AP: Apurinic/apyrimidinic (site)
APT: Adenine phosphoribosyltransferase
BER: Base excision repair
CDS: Coding DNA sequence
DSB(s): DNA double-strand break(s)
2-FA: 2-Fluoroadenine
HR: Homologous recombination
MMS: Methyl methanesulfonate
NHEJ: Nonhomologous end joining
N/N: Neutral comet assay
ROS: Reactive oxygen species
SCGE: Comet assay
SSB(s): DNA single-strand break(s)
*τ*_1/2_: Half-life.
