# Supplementary material for: Genotoxin Induced Mutagenesis in the Model Plant Physcomitrella patens
Source: Biomed Res Int. 2013 Dec 9;2013:535049. doi: 10.1155/2013/535049 (PMC3872018; doi:10.1155/2013/535049)
Supplement: Supplementary file 1 — Supplementary Figure 1: Normalized mutation frequencies in Physcomitrella wild type and lig4, ku70, rad50, mre11and nbs1 mutant lines. Supplementary Figure 2: Map and localization of APT primers used for rescue of APT locus and sequencing and which are listed in Supplemental Table1. Supplementary Table 2: Mutations (location and description) identified in the APT locus of Physcomitrella wild type and lig4, mre11 and rad50 mutant lines. [file 535049.f1.pdf]

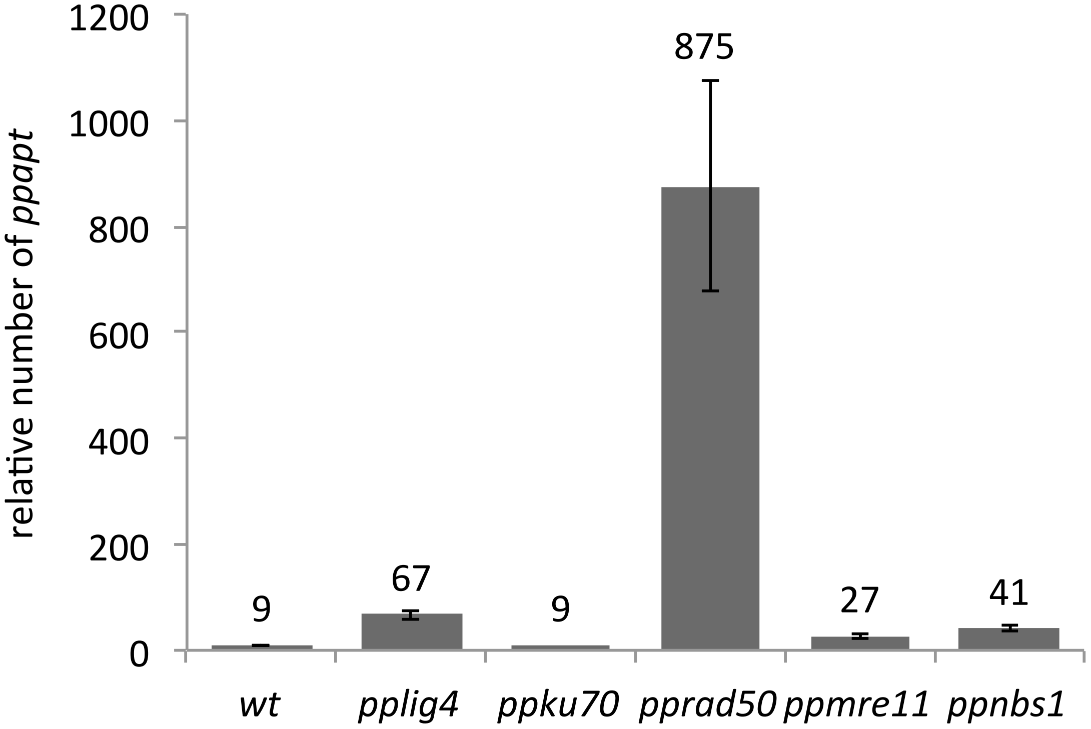

Supplemental Figure 1 Relative numbers of 2-FA resistant clones induced in *Physcomitrella* wild type and *lig4, ku70, rad50, mre11* and *nbs1* mutants by 1 µg mL^{-1} Bleomycin and normalized to 1 g dry tissue weight.

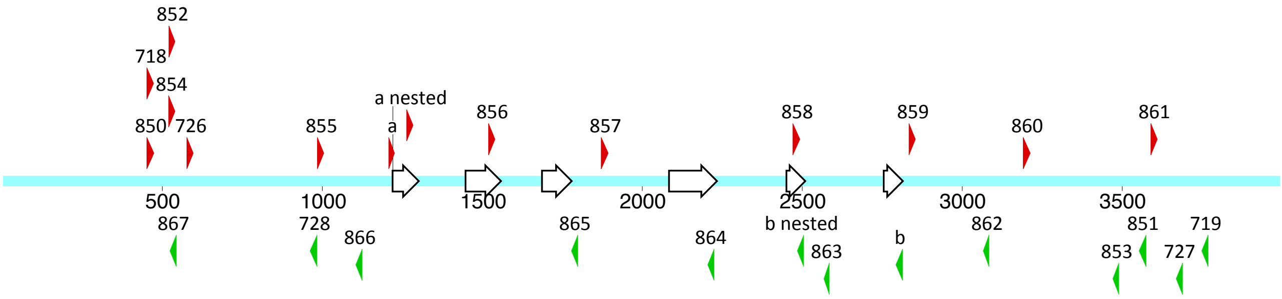

Supplemental Figure 2 Map and localization of *APT* primers listed in Supplemental Table 1. Turquoise line represents genomic sequence DQ117987 with indicated nucleotide numbers. Empty arrows indicate position of exons and red and green arrows, forward and reverse sequencing primers.

Primer #	Orientation	Sequence
a	F	CAGGAAGTGAAGATGTCG
a nested	F	GGACAGCATCCGTACCATTC
718	F	ATAAACAACGCGAGGATGAC
726	F	ATTCCACTCGCCGAAGGGAC
854	F	TTGGGTAACCGTGGAATCAG
855	F	CAGTGCCTGTGATGCGGTTG
856	F	TTGAGCGTTACCGGGACCAG
857	F	CAATGTGACCGAGACTTCATCC
858	F	GTGGAATGCGCGTGCTTGGTTG
859	F	GTCGCCGTGGTCATTGGTTC
860	F	GCCTCCTGATGTTTCTCTTACC
861	F	TCCACAAGGACCCAGTTACC
b	R	GGTGGAATATGAGGGCGAGTA
b nested	R	CTTGGTTGAGCTGGTAGACC
719	R	CAATCCACATCCAGCCAATG
727	R	CCGTCGAATCTGAACCGCGA
728	R	GAATTTGCTGTGGGTTATGGGC
851	R	GAATATCCTCACGTCGGAAAGC
853	R	GATGAGAAAGGAACTGCGC
862	R	CTTCTAACCTCGGAAACG
863	R	GAGTTCAATGCCGTCTGGAG
864	R	CACTCTTGGAGCTGCCATCA
865	R	GGTAAGGGTTGACAGGCACT
866	R	GGTCAATGTGGCAGCAAGTA
867	R	GTAACCGTGGAATCAGCAAC

Supplemental Table 1 Primers used for sequencing *APT* locus.

mutant line		INTRONS/NON CODING					EXONS				
	apt mutant #	substitution	point insertions	point detetions	insertions>2	deletions >2	substitution	point insertions	point detetions	insertions>2	deletions >2
wt	1	T-->A(1436)		A(1168)							
	2	T-->A(2173)<br>G-->T(2200)<br>T-->A(2361)		T(1800)	GCTT(2400)			G(1650)<br>T(1910)			
	3										1364:1372
	5						T-->A(747)				
	6			C(720)				G(752)			
pplig4	1		A(1138)								
	2		T(3683)								
	3		C(1108)					T(1240)			
	4	T-->A(2790)						T(790)			
ppmre11	1			G(715)					T(2619)		739:1125
	4			T(1847)			G-->T(2228)	T(2619)			
	6	G-->T(2871)									
pprad50	1							T(2213)			
	2	A-->T(1734)		T(1823)							1566:1571
	3						A-->T(1577)				1582:1637
	4		T-->A(1543)<br>A-->T(1544)<br>G-->T(1546)				A-->T(1204)<br>T-->A(1212)<br>T-->C(1407)<br>T-->A(1591)<br>T-->A(1604)<br>T-->C(1634)<br>C-->T(1869)		G(1640)		
	5								G(1640)		
	7							G(752)	T(761)		1166:1914

Supplemental Table 2 Mutations identified in the *APT* locus Pp1s114_124V6.1 (https://www.cosmoss.org) of 2-FA resistant clones induced by Bleomycin in *Physcomitrella* wild type and *lig4, mre11* and *rad50* mutants.
